# Two-loop QCD corrections to the MSSM Higgs masses beyond the effective-potential approximation

**DOI:** 10.1140/epjc/s10052-015-3280-5

**Published:** 2015-02-10

**Authors:** G. Degrassi, S. Di Vita, P. Slavich

**Affiliations:** 1 Dipartimento di Matematica e Fisica, Università di Roma Tre and INFN, Sezione di Roma Tre, Via della Vasca Navale 84, 00146 Rome, Italy; 2 Max-Planck-Institut für Physik, Föhringer Ring 6, 80805 Munich, Germany; 3 LPTHE, UPMC Univ. Paris 06, Sorbonne Universités, 4 Place Jussieu, 75252 Paris, France; 4 LPTHE, CNRS, 4 Place Jussieu, 75252 Paris, France

## Abstract

We compute the two-loop QCD corrections to the neutral Higgs-boson masses in the Minimal Supersymmetric Standard Model, including the effect of non-vanishing external momenta in the self-energies. We obtain corrections of ${\mathcal {O}}(\alpha _{t}\alpha _{s})$ and ${\mathcal {O}}(\alpha \alpha _{s})$, i.e., all two-loop corrections that involve the strong gauge coupling when the only non-vanishing Yukawa coupling is the top one. We adopt either the $\overline{\mathrm{DR}}$ renormalization scheme or a mixed on-shell (OS)–$\overline{\mathrm{DR}}$ scheme where the top/stop parameters are renormalized on-shell. We compare our results with those of earlier calculations, pointing out an inconsistency in a recent result obtained in the mixed OS–$\overline{\mathrm{DR}}$ scheme. The numerical impact of the new corrections on the prediction for the lightest-scalar mass is moderate, but already comparable to the accuracy of the Higgs-mass measurement at the Large Hadron Collider.

## Introduction

The accuracy of the measurement of the Higgs-boson mass by the ATLAS and CMS collaborations at the Large Hadron Collider (LHC) has already reached the level of 300–400 MeV [1, 2] and, being still dominated by statistics, is bound to improve further when the LHC restarts operations in 2015. This puts new emphasis on the need for high-precision calculations in those extensions of the Standard Model (SM), such as the Minimal Supersymmetric Standard Model (MSSM), in which the Higgs-boson mass can be predicted as a function of other physical observables.

The Higgs sector of the MSSM consists of two $SU(2)$ doublets, $H_1$ and $H_2$, whose relative contribution to electroweak (EW) symmetry breaking is determined by the ratio of vacuum expectation values (VEVs) of their neutral components, $\tan \beta \equiv v_2/v_1$. The spectrum of physical Higgs bosons is richer than in the SM, consisting of two neutral scalars, $h$ and $H$, one neutral pseudoscalar, $A$, and two charged scalars, $H^\pm$. At the tree level, the neutral scalar masses ${{m}_{h}}$ and ${{m}_{{\scriptscriptstyle H}}}$ and the scalar mixing angle $\alpha$ can be computed in terms of the $Z$-boson mass ${{m}_{{\scriptscriptstyle Z}}}$, the pseudoscalar mass ${{m}_{{\scriptscriptstyle A}}}$ and $\tan \beta$, and the bound ${{m}_{h}}< |\cos 2\beta |\,{{m}_{{\scriptscriptstyle Z}}}$ applies. In a significant portion of the parameter space the lightest scalar $h$ has SM-like couplings to fermions and gauge bosons, in which case the tree-level bound on ${{m}_{h}}$ has long been disproved by the LEP [3, 4]. However, radiative corrections can raise the prediction for the lightest-scalar mass up to the value ${{m}_{h}}\approx 125$ GeV observed at the LHC, and they bring along a dependence on all MSSM parameters. Among the latter, particularly relevant are the masses and mixing of the scalar partners of the third-generation quarks, the stop and sbottom squarks.

Due to the crucial role of radiative corrections in pushing the prediction for the lightest-scalar mass above the tree-level bound, an impressive theoretical effort has been devoted over more than 20 years to the precise determination of the Higgs sector of the MSSM.^1^ After the early realization [5–9] of the importance of the one-loop ${\mathcal {O}}(\alpha _{t})$ corrections^2^ involving top and stop, full one-loop computations of the MSSM Higgs masses have been provided [10–13], leading logarithmic effects at two loops have been included via renormalization-group methods [14–17], and genuine two-loop corrections of ${\mathcal {O}}(\alpha _{t}\alpha _{s})$ [18–25], ${\mathcal {O}}(\alpha _{t}^2)$ [18, 24, 26], ${\mathcal {O}}(\alpha _{b}\alpha _{s})$ [27, 28] and ${\mathcal {O}}(\alpha _{t}\alpha _{b}+ \alpha _{b}^2)$ [29] have been evaluated in the limit of vanishing external momentum in the Higgs self-energies. All of these corrections have been implemented in widely used computer codes for the calculation of the MSSM mass spectrum, such as FeynHiggs [30], SoftSUSY [31, 32], SuSpect [33] and SPheno [34, 35]. Furthermore, a complete two-loop calculation of the MSSM Higgs masses in the effective potential approach (i.e., at zero external momentum), including also two-loop corrections controlled by the EW gauge couplings, has been presented in Refs. [36, 37]. Some of the dominant three-loop corrections to ${{m}_{h}}$ have also been obtained, both via renormalization-group methods [38–40] and by explicit calculation of the Higgs self-energy at zero external momentum [41, 42].

Already at the two-loop level, going beyond the approximation of zero external momentum brings significant complications to the calculation of the Higgs self-energies. Different algorithms for expressing all two-loop self-energy integrals with arbitrary external momentum in terms of a minimal set of Master Integrals (MIs) were developed in Refs. [43–46]. However, explicit analytical formulas for the MIs can be derived only for special values of the masses of the particles circulating in the loops, whereas in the general case a numerical calculation becomes unavoidable. A method to compute all the MIs of Ref. [46] by numerically solving a system of differential equations in the external momentum was developed in Ref. [47], extending earlier results of Refs. [48–51]. A library of routines for the computation of the MIs with the method of Ref. [47] was then made available in the package TSIL [52].

A calculation of the two-loop contributions to the Higgs self-energies involving the strong gauge coupling or the third-family Yukawa couplings, based on the methods of Refs. [47, 52], was presented in Ref. [53]. That calculation goes beyond the two-loop results implemented in public codes [19–21, 25–29] in that it includes external-momentum effects, as well as contributions involving the D-term-induced EW interactions between Higgs bosons and sfermions. When combined with the effective-potential results of Refs. [36, 37], the results of Ref. [53] provide an almost-complete two-loop calculation of the Higgs masses in the MSSM – the only missing contributions being external-momentum effects that involve only the EW gauge couplings. However, no code for the calculation of the MSSM mass spectrum implementing the results of Refs. [36, 37, 53] was ever made available, and the way those results are organized does not lend itself to a straightforward implementation in the existing public codes. On one hand, the $\overline{\mathrm{DR}}$ renormalization scheme adopted in Refs. [36, 37, 53] for the parameters of the MSSM lagrangian does not match the “mixed on-shell (OS)–$\overline{\mathrm{DR}}$” scheme adopted in FeynHiggs. On the other hand, implementation of the results of Refs. [36, 37, 53] in SoftSUSY, SuSpect and SPheno, which also adopt the $\overline{\mathrm{DR}}$ scheme, is complicated by the fact that in Refs. [36, 37, 53] the running masses of the Higgs bosons entering the loop corrections are defined by the second derivatives of the tree-level potential. While this choice amounts to a legitimate reshuffling of terms between different perturbative orders, it restricts the applicability of the calculation to rather specific ranges of renormalization scale where none of the running Higgs masses – as defined in Refs. [36, 37, 53] – is tachyonic. Perhaps as a consequence of these complications, a full decade after the publication of Ref. [53] its results have yet to be included in phenomenological analyses of the MSSM Higgs sector.

In this paper we present a new calculation of the momentum-dependent part of the two-loop corrections to the neutral Higgs masses of ${\mathcal {O}}(\alpha _{t}\alpha _{s})$, i.e. those involving both the top Yukawa coupling and the strong gauge coupling. We also compute “mixed” two-loop corrections that we denote by ${\mathcal {O}}(\alpha \alpha _{s})$: they involve both the strong gauge coupling and the EW gauge couplings, under the approximation that the only non-vanishing Yukawa coupling is the top one. It is natural to consider these two classes of corrections together, because in both of them the dominant terms affecting the lightest-scalar mass are expected to be suppressed by a factor of $\mathcal{O}(m_Z^2/{{m}_{t}}^2)$ with respect to the zero-momentum ${\mathcal {O}}(\alpha _{t}\alpha _{s})$ corrections (in practice, we find that both classes of corrections are considerably more suppressed than that, but still comparable to each other in size).

In our calculation we rely on the integration-by-parts (IBP) technique of Refs. [54, 55] to express the momentum-dependent loop integrals in terms of the MIs of Ref. [46], which we evaluate by means of the package TSIL. We obtain results for both the $\overline{\mathrm{DR}}$ and the OS–$\overline{\mathrm{DR}}$ renormalization schemes, organized in such a way that they can be directly implemented in the existing codes for the computation of the MSSM mass spectrum. We verify that our results are in full agreement with the ones of Ref. [53] where they overlap. After describing our calculation in some detail, we briefly discuss the numerical impact of the momentum-dependent ${\mathcal {O}}(\alpha _{t}\alpha _{s})$ and ${\mathcal {O}}(\alpha \alpha _{s})$ corrections to the Higgs masses in a set of representative points in the MSSM parameter space.

While our paper was in preparation, an independent calculation of the momentum-dependent ${\mathcal {O}}(\alpha _{t}\alpha _{s})$ corrections to the neutral Higgs masses in the MSSM appeared [56], relying on the results of Ref. [43] for the decomposition of two-loop integrals into MIs and on the package SecDec [57, 58] for the numerical evaluation of the latter. The results of that calculation are expressed in the OS–$\overline{\mathrm{DR}}$ scheme, and they have been implemented in the latest version of FeynHiggs. Although we have verified that our results for the contributions of genuine two-loop diagrams involving the strong-gauge and top-Yukawa couplings agree numerically with those of Ref. [56], we do not reproduce the overall values of the momentum-dependent ${\mathcal {O}}(\alpha _{t}\alpha _{s})$ corrections to the Higgs masses. We trace the reason for the discrepancy to an inconsistency in Ref. [56] concerning the definitions of the wave-function-renormalization (WFR) constants for the Higgs fields and of the parameter $\tan \beta$.

## Neutral Higgs boson masses in the MSSM

We outline here our calculation of the two-loop corrections to the masses of the neutral Higgs bosons in the MSSM with real parameters (we do not consider the possibility of CP violation in the Higgs sector). We decompose the neutral components of the two Higgs doublets into their VEVs plus their CP-even and CP-odd fluctuations as follows:

1 $$\begin{aligned}&H_1^0 \equiv \frac{1}{\sqrt{2}}\,(v_1 + S_1 + i \, P_1), \nonumber \\&H_2^0 \equiv \frac{1}{\sqrt{2}}\,(v_2 + S_2 + i \, P_2). \end{aligned}$$

The CP-odd components $P_1$ and $P_2$ combine into the pseudoscalar $A$ and the neutral would-be Goldstone boson $G^0$. The CP-even components $S_1$ and $S_2$ combine into the scalars $h$ and $H$. The squared physical masses of the latter are the two solutions for $p^2$ of the equation

2 $$\begin{aligned} \mathrm{det} \left[ \Gamma _S(p^2) \right] = 0, \end{aligned}$$

where $\Gamma _S(p^2)$ denotes the $2\times 2$ inverse-propagator matrix in the $(S_1,S_2)$ basis, $p$ being the external momentum flowing into the scalar self-energies. We can decompose $\Gamma _S(p^2)$ as

3 $$\begin{aligned} \Gamma _S(p^2) = p^2 - \mathcal{M}_0^2 - \Delta \mathcal{M}^2(p^2), \end{aligned}$$

where $\mathcal{M}_0^2$ denotes the tree-level mass matrix written in terms of renormalized parameters, and $\Delta \mathcal{M}^2(p^2)$ collectively denotes the radiative corrections. At each order in the perturbative expansion, the latter include both the contributions of one-particle-irreducible (1PI) diagrams and non-1PI counterterm contributions arising from the renormalization of parameters that enter the lower-order parts of $\Gamma _S(p^2)$. We express $\mathcal{M}_0^2$ in terms of the parameter $\tan \beta$ renormalized in the $\overline{\mathrm{DR}}$ scheme, and of the physical masses of the pseudoscalar and of the $Z$ boson

4 $$\begin{aligned} \mathcal{M}_0^2 = \left( \begin{array}{cc} c^2_\beta \,m_Z^2 + s^2_\beta \,m_A^2 &{}\quad - {{s}_{\beta }}\, {{c}_{\beta }}\left( m_Z^2 + m_A^2 \right) \\ - {{s}_{\beta }}\, {{c}_{\beta }}\left( m_Z^2 + m_A^2\right) &{}\quad s^2_\beta \, m_Z^2 + c^2_\beta \, m_A^2 \end{array}\right) , \end{aligned}$$

using (here and thereafter) the shortcuts $c_\phi \equiv \cos \phi$ and $s_\phi \equiv \sin \phi$ for a generic angle $\phi$. Neglecting terms that do not contribute at ${\mathcal {O}}(\alpha _{t}\alpha _{s})$ or ${\mathcal {O}}(\alpha \alpha _{s})$, our choices for the parameters entering $\mathcal{M}_0^2$ lead to the following expressions for the two-loop parts of the individual entries of $\Delta \mathcal{M}^2(p^2)$:

5 $$\begin{aligned} \left[ \Delta \mathcal{M}^2(p^2)\right] ^{(2)}_{11}&= s_\beta ^2\, \mathrm{Re}\, \Pi _{{\scriptscriptstyle A}{\scriptscriptstyle A}}^{(2)}(m_A^2) + c_\beta ^2\,\mathrm{Re}\, \Pi _{{\scriptscriptstyle Z}{\scriptscriptstyle Z}}^{(2)}(m_Z^2)\nonumber \\&-\Pi _{11}^{(2)}(p^2) -\delta \mathcal {Z}_1^{(2)} \left( p^2- c^2_\beta \, m_Z^2 - s^2_\beta \,m_A^2\right) \nonumber \\&+ \left( 1- {{s}_{\beta }}^4\right) \frac{~\,T_1^{(2)}}{v_1} - s_\beta ^2 c_\beta ^2 \frac{~\,T_2^{(2)}}{v_2}\nonumber \\&-2\,s_\beta ^2 c_\beta ^2\, \left( m_Z^2-m_A^2\right) \frac{~\delta \tan \beta ^{(2)}}{\tan \beta } , \end{aligned}$$

6 $$\begin{aligned} \left[ \Delta \mathcal{M}^2(p^2)\right] ^{(2)}_{12}&= - {{s}_{\beta }}{{c}_{\beta }}\left[ \mathrm{Re}\, \Pi _{{\scriptscriptstyle A}{\scriptscriptstyle A}}^{(2)}(m_A^2) + \mathrm{Re}\, \Pi _{{\scriptscriptstyle Z}{\scriptscriptstyle Z}}^{(2)}(m_Z^2)\right. \nonumber \\&-\left. s_\beta ^2 \frac{~\,T_1^{(2)}}{v_1} - c_\beta ^2 \frac{~\,T_2^{(2)}}{v_2} \right] -\Pi _{12}^{(2)}(p^2)\nonumber \\&-\frac{1}{2}{{s}_{\beta }}{{c}_{\beta }}\left( m_Z^2 + m_A^2\right) \nonumber \\&\times \left[ 2\, (c_\beta ^2-s_\beta ^2)\frac{~\delta \tan \beta ^{(2)}}{\tan \beta }\!+\! \delta \mathcal {Z}_1^{(2)}\!+\!\delta \mathcal {Z}_2^{(2)}\!\right] ,\nonumber \\ \end{aligned}$$

7 $$\begin{aligned} \left[ \Delta \mathcal{M}^2(p^2)\right] ^{(2)}_{22}&= c_\beta ^2\, \mathrm{Re}\, \Pi _{{\scriptscriptstyle A}{\scriptscriptstyle A}}^{(2)}(m_A^2) + s_\beta ^2 \, \mathrm{Re}\, \Pi _{{\scriptscriptstyle Z}{\scriptscriptstyle Z}}^{(2)}(m_Z^2)\nonumber \\&-\Pi _{22}^{(2)}(p^2) -\delta \mathcal {Z}_2^{(2)} \left( p^2- s^2_\beta \,m_Z^2 - c^2_\beta \, m_A^2 \right) \nonumber \\&- s_\beta ^2 c_\beta ^2 \frac{~\,T_1^{(2)}}{v_1} + \left( 1- {{c}_{\beta }}^4\right) \frac{~\,T_2^{(2)}}{v_2}\nonumber \\&+2\,s_\beta ^2c_\beta ^2\, \left( m_Z^2-m_A^2\right) \frac{~\delta \tan \beta ^{(2)}}{\tan \beta }. \end{aligned}$$

In the equations above, $T^{(2)}_i$ and $\Pi ^{(2)}_{ij}$ (with $i,j=1,2$) denote the two-loop parts of tadpoles and self-energies, respectively, for the scalars $S_i$, while $\Pi ^{(2)}_{{\scriptscriptstyle A}{\scriptscriptstyle A}}$ and $\Pi ^{(2)}_{{\scriptscriptstyle Z}{\scriptscriptstyle Z}}$ denote the two-loop parts of the pseudoscalar and $Z$-boson self-energies. In addition, $\delta \mathcal{Z}^{(2)}_i$ (with $i=1,2$) in Eqs. (5)–(7) denote the two-loop parts of the WFR counterterms for the Higgs fields $H^0_i$, which we renormalize as follows:

8 $$\begin{aligned} H^0_i ~\longrightarrow ~\sqrt{\mathcal{Z}_i}\,H^0_i ~\simeq ~ \left( 1 + \frac{1}{2}\,\delta \mathcal{Z}^{(1)}_i + \frac{1}{2}\,\delta \mathcal{Z}^{(2)}_i \right) \,H^0_i, \end{aligned}$$

where in the expansion of the square root we have again neglected terms that do not contribute at ${\mathcal {O}}(\alpha _{t}\alpha _{s})$ or ${\mathcal {O}}(\alpha \alpha _{s})$. We adopt a $\overline{\mathrm{DR}}$ definition for the $\mathcal{Z}_i$, which can then be determined from the anomalous dimensions of the Higgs fields and from the $\beta$ functions of the couplings entering the anomalous dimensions. Taking from the general formulas of Refs. [59, 60] only the terms relevant to our approximation, we get

9 $$\begin{aligned} \delta \mathcal{Z}^{(1)}_1&= \delta \mathcal{Z}^{(2)}_1= 0,\quad \delta \mathcal{Z}^{(1)}_2 = - \frac{\alpha _{t}}{4\pi }\,N_c\,\cdot \,\frac{1}{\epsilon },\nonumber \\ \delta \mathcal{Z}^{(2)}_2&= \frac{\alpha _{t}\alpha _{s}}{(4\pi )^2}\,2\,N_c\,C_F\cdot \left( \frac{1}{\epsilon ^2}-\frac{1}{\epsilon }\right) , \end{aligned}$$

where $N_c=3$ and $C_F=4/3$ are color factors, $\epsilon =(4-d)/2\,$ in dimensional reduction, and the coupling $\alpha _{t}$ entering the one-loop counterterm $\delta \mathcal{Z}^{(1)}_2$ is in turn renormalized in the $\overline{\mathrm{DR}}$ scheme. Finally, $\delta \tan \beta ^{(2)}$ in Eqs. (5)–(7) denotes the two-loop part of the counterterm for the parameter $\tan \beta$. The choice of a $\overline{\mathrm{DR}}$ definition for $\tan \beta$ implies that, in our approximation, its counterterm can be expressed via the WFR counterterms:

10 $$\begin{aligned} \frac{~\delta \tan \beta ^{(\ell )}}{\tan \beta } =\frac{1}{2}\,\left( \delta \mathcal{Z}^{(\ell )}_2 -\delta \mathcal{Z}^{(\ell )}_1\right) \quad (\ell =1,2). \end{aligned}$$

All tadpoles and self-energies in Eqs. (5)–(7) include both 1PI two-loop contributions and non-1PI contributions arising from the renormalization of the parameters entering their one-loop counterparts. Since we are focusing on the ${\mathcal {O}}(\alpha _{t}\alpha _{s})$ and ${\mathcal {O}}(\alpha \alpha _{s})$ corrections to the Higgs masses, we need to introduce counterterms only for the parameters that are subject to ${\mathcal {O}}(\alpha _{s})$ corrections, namely the top mass $m_t$, the stop masses $m_{\tilde{t}_{1}}$ and $m_{\tilde{t}_{2}}$, the stop mixing angle $\theta _t$, the soft supersymmetry-breaking Higgs-stop coupling $A_t$ and the masses $m_{\tilde{q}_i}$ of all squarks other than the stops. The latter enter the one-loop tadpoles and self-energies of the Higgs bosons via D-term-induced EW couplings, and the one-loop self-energy of the $Z$ boson via the gauge interaction. In our calculation we neglect all Yukawa couplings (and hence quark masses) other than the top one,^3^ therefore none of the other squarks mix. We obtained results for the ${\mathcal {O}}(\alpha _{t}\alpha _{s})$ and ${\mathcal {O}}(\alpha \alpha _{s})$ contributions to tadpoles and self-energies assuming that the relevant quark/squark parameters are renormalized either in the $\overline{\mathrm{DR}}$ or in the OS scheme. Formulas for the $\overline{\mathrm{DR}}$–OS shift of the parameters in the top/stop sector can be found, e.g., in appendix B of Ref. [25], while the shifts for the remaining squark masses can be obtained by setting ${{m}_{t}}=\theta _t=0$ in the corresponding formulas for the stop masses. We remark that the right-hand sides of Eqs. (5)–(7) are constructed to give finite entries in the inverse-propagator matrix of the scalars. Indeed we have explicitly verified that – after summing all 1PI and counterterm contributions – the $1/\epsilon ^2$ and $1/\epsilon$ poles in the right-hand sides of Eqs. (5)–(7) cancel out.

In principle, the two-loop contributions to the Higgs inverse propagator given in Eqs. (5)–(7) must be combined with a full calculation of the corresponding one-loop contributions, and used to determine the physical Higgs masses by solving directly Eq. (2). However, as will be discussed in the next section, the computing times required for the evaluation of momentum-dependent two-loop integrals are not negligible. A numerical search for the solutions of Eq. (2) could significantly slow down the codes for the calculation of the Higgs masses, making them unsuitable for extensive phenomenological analyses of the MSSM parameter space. It is therefore convenient to compute the Higgs masses in two steps, with a procedure similar to the one discussed in Refs. [10, 26]. We first call FeynHiggs, which solves Eq. (2) including in $\Delta \mathcal{M}^2(p^2)$ the full one-loop corrections plus the dominant two-loop corrections of ${\mathcal {O}}(\alpha _{t}\alpha _{s})$, ${\mathcal {O}}(\alpha _{b}\alpha _{s})$, and $\mathcal{O}(\alpha _{t}^2+\alpha _{t}\alpha _{b}+\alpha _{b}^2)$ computed in the approximation of vanishing external momentum. From FeynHiggs we obtain the scalar masses $\overline{m}_h^2$ and $\overline{m}_{{\scriptscriptstyle H}}^2$, and an effective mixing angle $\overline{\alpha }$ which diagonalizes the loop-corrected scalar mass matrix at vanishing external momentum. Our full results for the scalar masses are then obtained by adding to the results of FeynHiggs the momentum-dependent parts of the ${\mathcal {O}}(\alpha _{t}\alpha _{s})$ corrections and the whole ${\mathcal {O}}(\alpha \alpha _{s})$ corrections:

11 $$\begin{aligned} m^2_{h,{\scriptscriptstyle H}} = \overline{m}^2_{h,{\scriptscriptstyle H}} ~+~ (\Delta m^2_{h,{\scriptscriptstyle H}})^{\alpha _{t}\alpha _{s},\,p^2} ~+~ (\Delta m^2_{h,{\scriptscriptstyle H}})^{\alpha \alpha _{s}}. \end{aligned}$$

Concerning the former, we have

12 $$\begin{aligned} \left( \Delta m^2_{h}\right) ^{\alpha _{t}\alpha _{s},\,p^2}&= c^2_{\beta -\overline{\alpha }}\,\Delta \Pi _{{\scriptscriptstyle A}{\scriptscriptstyle A}}^{(2)}\left( m_A^2\right) - s^2_{\overline{\alpha }}\, \Delta \Pi _{11}^{(2)}\left( \overline{m}_h^2\right) \nonumber \\&\quad + s_{2\overline{\alpha }}\,\Delta \Pi _{12}^{(2)}\left( \overline{m}_h^2\right) - c^2_{\overline{\alpha }}\,\Delta \Pi _{22}^{(2)}\left( \overline{m}_h^2\right) , \end{aligned}$$

13 $$\begin{aligned} \left( \Delta m^2_{{\scriptscriptstyle H}}\right) ^{\alpha _{t}\alpha _{s},\,p^2}&= s^2_{\beta -\overline{\alpha }}\,\Delta \Pi _{{\scriptscriptstyle A}{\scriptscriptstyle A}}^{(2)}\left( m_A^2\right) - c^2_{\overline{\alpha }}\, \Delta \Pi _{11}^{(2)}\left( \overline{m}_{{\scriptscriptstyle H}}^2\right) \nonumber \\&\quad - s_{2\overline{\alpha }}\,\Delta \Pi _{12}^{(2)}\left( \overline{m}_{{\scriptscriptstyle H}}^2\right) - s^2_{\overline{\alpha }}\,\Delta \Pi _{22}^{(2)}\left( \overline{m}_{{\scriptscriptstyle H}}^2\right) , \end{aligned}$$

where we define $\Delta \Pi (p^2) \,\equiv \, \Pi (p^2)-\Pi (0)$, and retain only the real and finite part of the ${\mathcal {O}}(\alpha _{t}\alpha _{s})$ contributions to the two-loop self-energies. For what concerns the ${\mathcal {O}}(\alpha \alpha _{s})$ corrections, they contain all terms from Eqs. (5)–(7):

14 $$\begin{aligned} \left( \Delta m^2_{h}\right) ^{\alpha \alpha _{s}}&= s^2_{\overline{\alpha }}\,\left[ \Delta \mathcal{M}^2\left( \overline{m}_h^2\right) \right] ^{\alpha \alpha _{s}}_{11}\nonumber \\&\quad - s_{2\overline{\alpha }}\,\left[ \Delta \mathcal{M}^2\left( \overline{m}_h^2\right) \right] ^{\alpha \alpha _{s}}_{12} + c^2_{\overline{\alpha }}\,\left[ \Delta \mathcal{M}^2\left( \overline{m}_h^2\right) \right] ^{\alpha \alpha _{s}}_{22},\end{aligned}$$

15 $$\begin{aligned} \left( \Delta m^2_{{\scriptscriptstyle H}}\right) ^{\alpha \alpha _{s}}&= c^2_{\overline{\alpha }}\,\left[ \Delta \mathcal{M}^2\left( \overline{m}_{{\scriptscriptstyle H}}^2\right) \right] ^{\alpha \alpha _{s}}_{11}\nonumber \\&\quad +\, s_{2\overline{\alpha }}\,\left[ \Delta \mathcal{M}^2\left( \overline{m}_{{\scriptscriptstyle H}}^2\right) \right] ^{\alpha \alpha _{s}}_{12} + s^2_{\overline{\alpha }}\,\left[ \Delta \mathcal{M}^2\left( \overline{m}_{{\scriptscriptstyle H}}^2\right) \right] ^{\alpha \alpha _{s}}_{22}, \end{aligned}$$

where again we take the real part of all two-loop self-energies. We remark that Ref. [56] proposes an alternative two-step procedure to include the momentum-dependent parts of the ${\mathcal {O}}(\alpha _{t}\alpha _{s})$ corrections in FeynHiggs, differing from the one outlined above only by higher-order effects.

Finally, a comment is in order about the dependence of the corrections to the Higgs masses on the WFR constants.^4^ In principle, the predictions for the physical Higgs masses at a given order in the perturbative expansion should not depend directly on the WFR constants (although they could still depend indirectly on them via the $\tan \beta$ counterterm). Indeed, if the two-loop contributions to the inverse-propagator matrix are computed with $p^2$ equal to the tree-level scalar masses and then rotated to the mass-eigenstate basis via the tree-level mixing angle, so that the computation is performed strictly at the two-loop level, the terms in Eqs. (5)–(7) that depend explicitly on $\delta \mathcal{Z}^{(2)}_i$ drop out of the mass corrections $\Delta m^2_{h,{\scriptscriptstyle H}}\,$. On the other hand, if the loop-corrected scalar masses $\overline{m}_h^2$ and $\overline{m}_{{\scriptscriptstyle H}}^2$ and the effective mixing angle $\overline{\alpha }$ are used, as in Eqs. (12)–(15) above, or if the zeroes of the inverse-propagator matrix are determined numerically, the corrections to the scalar masses retain a dependence on the WFR counterterms. In our calculation we adopt a $\overline{\mathrm{DR}}$ definition for the WFR; therefore the terms involving $\delta \mathcal{Z}^{(2)}_i$ are purely divergent and cancel out against other divergent terms in the individual entries of the inverse-propagator matrix, hence they do not show up in Eqs. (12) and (13). If, however, one adopts a non-minimal definition of the WFR, Higgs-mass corrections computed as in Eqs. (14) and (15) will contain non-vanishing terms that depend on the finite part of $\delta \mathcal{Z}^{(2)}_i$. Albeit formally of higher order in the perturbative expansion, these terms can be numerically relevant when the loop-corrected scalar masses differ significantly from their tree-level values (as is the case for a SM-like scalar $h$ with mass around 125 GeV).

## Calculation of two-loop diagrams with nonzero momentum

The computation of the two-loop corrections to the neutral MSSM Higgs masses considered in this paper requires the knowledge of the tadpole and self-energy diagrams entering Eqs. (5)–(7). While the strategy for the computation in the zero-momentum approximation is well known, the evaluation of the self-energies with arbitrary external momentum is more involved. We illustrate in this section the details of our calculation, which we performed in a fully automated way.

The relevant diagrams are generated with FeynArts [67], using a modified version of the original MSSM model file [68] that implements the QCD interactions in the background field gauge. The diagrams contributing to the vacuum polarization of the $Z$ boson are contracted with a suitable projector in order to single out their transverse part. The color factors are simplified with a private package and the Dirac algebra is handled by FORM [69]. The computation is performed in dimensional reduction, which we can implement in this case by generating the diagrams in dimensional regularization and replacing, in each diagram involving an internal $d$-dimensional gluon, $g^{\mu \nu } \rightarrow g^{\mu \nu } + g^{\hat{\mu }\hat{\nu }}$ (where $g^{\hat{\mu }\hat{\nu }}$ is the $2\epsilon$-dimensional metric tensor) in order to include the corresponding $\epsilon$-scalar contribution. We are then left with Feynman integrals of the form

16 $$\begin{aligned} \int d^dk_1 d^dk_2 \frac{\left( k_1^2\right) ^\alpha \left( k_2^2\right) ^\beta (k_1\cdot p)^\gamma (k_2\cdot p)^\delta (k_1\cdot k_2)^\eta }{D_1^{a_1} D_2^{a_2} D_3^{a_3} D_4^{a_4} D_5^{a_5}}, \end{aligned}$$

where $\alpha , \ldots , \eta ,\, a_1, \ldots , a_5$ are positive (or zero) integer exponents and the $D_i$’s are defined as

$$\begin{aligned} D_1&= k_1^2 - m_1^2, \, D_2 = (k_1-p)^2 - m_2^2, \, D_3 = k_2^2 - m_3^2,\\ D_4&= (k_2-p)^2 - m_4^2, \, D_5 = (k_1-k_2)^2 - m_5^2. \end{aligned}$$

Integrals belonging to the class above are in general not linearly independent of each other. When the scalar products in the numerator are expressed in terms of the denominators, powers of a $D_i$ present in the original integral might cancel against a $D_i$ in the numerator, possibly generating a Feynman integral in which $D_i$ does not appear, i.e. in which the corresponding line has been shrunk to a point. For given $a_i$’s and high enough $\alpha ,\ldots ,\eta$, some $D_i$’s may acquire negative exponents. The computation of a Feynman integral of the type in Eq. (16) therefore reduces to the evaluation of a number of integrals of the form

17 $$\begin{aligned} \int \frac{d^dk_1 d^dk_2}{D_1^{n_1} D_2^{n_2} D_3^{n_3} D_4^{n_4} D_5^{n_5}}, \end{aligned}$$

where the exponents $n_i\in \mathbb {Z}$. In the present case, one has to evaluate $\mathcal {O}(300)$ different Feynman integrals.

There exists a convenient procedure for dealing with such large numbers of different Feynman integrals in a more efficient way than their direct evaluation. Dimensionally regularized integrals, at arbitrary loop order and with arbitrary number of external legs, satisfy identities of the IBP type [54, 55]. These identities are linear relations that connect integrals with different sets of exponents $\{n_1,\ldots ,n_5\}$. After a set of independent integrals, the MIs, has been identified, all the remaining integrals can then be reduced to linear combinations of the MIs, the coefficients being rational functions of the masses, the kinematic invariants and the space-time dimension $d$. One practical advantage of such a procedure is its “divide and conquer” spirit. On the one hand few MIs encode the analyticity properties (singularities, thresholds, branch cuts) of the problem under consideration. On the other hand, the evaluation of the large number of different Feynman integrals entering a computation is reduced to a problem of linear algebra, which can easily be automated, if the MIs are known. We perform the reduction to MIs with the public code REDUZE [70, 71], which implements the Laporta algorithm [72] and produces the IBP identities relevant to our case.

The evaluation of the MIs is in general a complicated problem and can proceed via different techniques, like the integration in the Feynman, Schwinger or Mellin–Barnes representations. A remarkable consequence of the aforementioned IBP relations is that the MIs obey linear systems of first-order differential equations (DEs) in the kinematic invariants, which provide an alternative means for their computation [73, 74]. Finding the analytic solution of the DEs for arbitrary $d$ is possible only in some simple cases. In more general cases the MIs are expanded in powers of $\epsilon = (4-d)/2$, giving rise to a (generally coupled) system of DEs for the expansion coefficients. In the limit of vanishing external momentum, two-loop self-energies become two-loop vacuum diagrams, which reduce via IBP to linear combinations of only one genuine two-loop MI and products of the one-loop one-propagator MI [75]. Two-loop self-energies with arbitrary external momentum, in the general case with five different masses in the loops, reduce via IBP to linear combinations of 30 MIs [46]. The finite part of such MIs can be expressed in terms of four functions, in addition to the well-known one-loop MIs.^5^ As already mentioned, analytic solutions for such functions have been derived only for special patterns of up to two internal masses (only one function is known in a particular case with three different masses). On the other hand, the diagrams that in our approximation contribute to the self-energies entering Eqs. (5)–(7) require the knowledge of MIs with up to four different masses, the most complicated ones being those involving simultaneously $m_t^2,{m}_{\tilde{t}_1}^{2},{m}_{\tilde{t}_2}^{2}$, and $m_{\tilde{g}}^{2}$.

In our computation we rely on the package TSIL [52], which implements (besides all the analytically known cases) the numerical solution of the DEs for the two-loop self-energy MIs. The method of Refs. [47–51] is based on the fact that the value at $p^2=0$ (or the expansion for small $p^2$ in the case of logarithmically divergent integrals) is known for each function and can be used to build the set of initial conditions needed for the solution of the DEs. In the computation of the self-energies entering Eqs. (5)–(7) we need to evaluate the corresponding MIs at $p^2=m_Z^2,m_A^2,m_h^2,m_H^2$. Given that we include the contribution of light quarks to $\Pi _{{\scriptscriptstyle Z}{\scriptscriptstyle Z}}$ (in the approximation $m_q=0$) and that ${{m}_{{\scriptscriptstyle A}}}$ is a free parameter, it is clear that the way thresholds are handled in the numerical evaluation of the MIs is of particular relevance. In the DEs approach, the physical two- and three-particle thresholds show up, together with the pseudothresholds, as poles in the coefficients of the DEs. TSIL overcomes the numerical instabilities related to such poles by displacing the $p^2$-integration contour in the upper half-plane when the momentum is above the smallest (pseudo)threshold. The evaluation at (or very close to) the (pseudo)thresholds is performed through a variant of the algorithm, which is slightly less efficient but ensures reliable results in such critical cases. As an example, the time needed on an Intel Core i7-4650U CPU for the evaluation of the *complete set* of MIs, for any of the mass patterns entering the self-energies, ranges between $5 \times 10^{-4}\,s$ and $8 \times 10^{-2}\,s$, the latter being the typical time for $\sqrt{p^2}$ close or equal to the heavy stop pair threshold and to the three-particle (pseudo)thresholds $m_{\tilde{t}_i}+m_{\tilde{g}}\pm {{m}_{t}}$. In Ref. [52] the relative accuracy of TSIL is claimed to be better than $10^{-10}$ for generic cases, or worse in cases with large mass hierarchies. Being TSIL a package dedicated to the evaluation of the MIs for two-loop self-energy diagrams, it is not surprising that its speed and accuracy prove much better than those quoted in Ref. [56], where the general-purpose package SecDec is used and, in the most complicated case, $100\,s$ are needed in order to reach a relative accuracy of at least $10^{-5}$ close to a threshold.

## Numerical examples

In this section we assess the numerical impact of the momentum-dependent part of the ${\mathcal {O}}(\alpha _{t}\alpha _{s})$ corrections and of the whole ${\mathcal {O}}(\alpha \alpha _{s})$ corrections on the predictions for the neutral Higgs-boson masses in the MSSM. We focus on six benchmark scenarios introduced in Ref. [77], which identify regions in the MSSM parameter space compatible with the current bounds from SUSY-particle searches and with the requirement that the predicted value of ${{m}_{h}}$ agrees, within the theoretical uncertainty of $\pm 3~\mathrm{GeV}$ estimated in Refs. [78, 79], with the mass of the SM-like Higgs boson discovered at the LHC.^6^

In our numerical examples we adopt the mixed OS–$\overline{\mathrm{DR}}$ scheme described in Sect. 2. The SM input parameters are chosen as the pole top mass $m_t = 173.2~\mathrm{GeV}$, the running bottom mass $m_b(m_b) = 4.2~\mathrm{GeV}$, the Fermi constant $G_F = 1.16639 \times 10^{-5}~\mathrm{GeV}^{-2}$, the strong gauge coupling $\alpha _s({{m}_{{\scriptscriptstyle Z}}}) = 0.118$, and the pole gauge-boson masses ${{m}_{{\scriptscriptstyle Z}}}= 91.1876~\mathrm{GeV}$ and ${{m}_{{\scriptscriptstyle W}}}= 80.385~\mathrm{GeV}$. To compute the scalar masses $\overline{m}^2_{h,{\scriptscriptstyle H}}$ and the effective mixing angle $\overline{\alpha }$ entering the corrections in Eqs. (11)–(15), we call FeynHiggs version 2.10.2. We use default values for all settings with the exception of runningMT = 0, i.e. the top mass in the radiative corrections is identified with the pole mass (to match the renormalization conditions imposed both in our OS–$\overline{\mathrm{DR}}$ calculation and in the one of Ref. [56]). By default, the renormalization scale associated to the $\overline{\mathrm{DR}}$ definition of the Higgs WFR and of $\tan \beta$ is fixed as $\mu _{{\scriptscriptstyle R}}=m_t$.

**Fig. 1:**
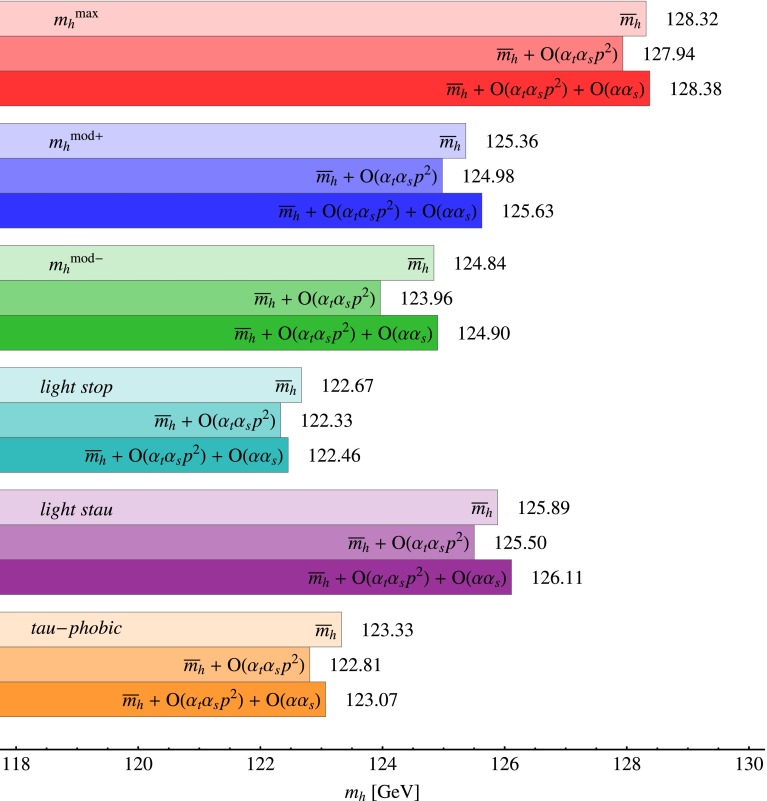
Predictions for the mass of the lightest scalar $h$ in the six benchmark scenarios of Ref. [77], for ${{m}_{{\scriptscriptstyle A}}}=500~\mathrm{GeV}$ and $\tan \beta =20$. For each scenario, the *three bars* show: the “unperturbed” mass $\overline{m}_h$ computed with FeynHiggs 2.10.2 (*upper*), the inclusion of the momentum-dependent part of the ${\mathcal {O}}(\alpha _{t}\alpha _{s})$ corrections (*middle*) and the additional inclusion of the whole ${\mathcal {O}}(\alpha \alpha _{s})$ corrections (*lower*). From *top* to *bottom*, the considered scenarios are $m_h^{\mathrm{max}}$ (*red*), ${{m}_{h}}^\mathrm{mod+}$ (*blue*), ${{m}_{h}}^\mathrm{mod-}$ (*green*), *light stop* (*turquoise*), *light stau* (*purple*), *tau-phobic* (*orange*)

In Fig. 1 we present our predictions for the lightest-scalar mass $m_h$ in the six benchmark scenarios. We choose ${{m}_{{\scriptscriptstyle A}}}= 500~\mathrm{GeV}$ and $\tan \beta =20$, so that the lightest scalar $h$ is SM-like, the bound on its tree-level mass is saturated, and the corrections controlled by the bottom Yukawa coupling, which we do not compute beyond the approximations of FeynHiggs, are not expected to be particularly relevant. For each scenario we show three bars: the upper one represents the “unperturbed” mass $\overline{m}_{h}$, obtained from FeynHiggs without additional corrections; the middle bar includes the effect of the momentum-dependent part of the ${\mathcal {O}}(\alpha _{t}\alpha _{s})$ corrections, i.e. the $(\Delta m_h^2)^{\alpha _{t}\alpha _{s},\,p^2}$ defined in Eq. (12); finally, the lower bar represents our final result for $m_h$, and includes the effects of both the momentum-dependent part of the ${\mathcal {O}}(\alpha _{t}\alpha _{s})$ corrections and the ${\mathcal {O}}(\alpha \alpha _{s})$ corrections, i.e. the $(\Delta m_h^2)^{\alpha \alpha _{s}}$ defined in Eq. (14).

Figure 1 shows that, in all considered scenarios, the momentum-dependent part of the ${\mathcal {O}}(\alpha _{t}\alpha _{s})$ corrections and the whole ${\mathcal {O}}(\alpha \alpha _{s})$ corrections can shift the prediction for $m_h$ by several hundred MeV each (the largest shifts, of about $\pm 1$ GeV, occur in the $m_h^\mathrm{mod-}$ scenario). However, in all of our examples the two classes of corrections happen to be similar to each other in magnitude, and to enter the prediction for $m_h$ with opposite signs. As a result, their combined effect is always fairly small, less than $\pm 300$ MeV.

**Fig. 2:**
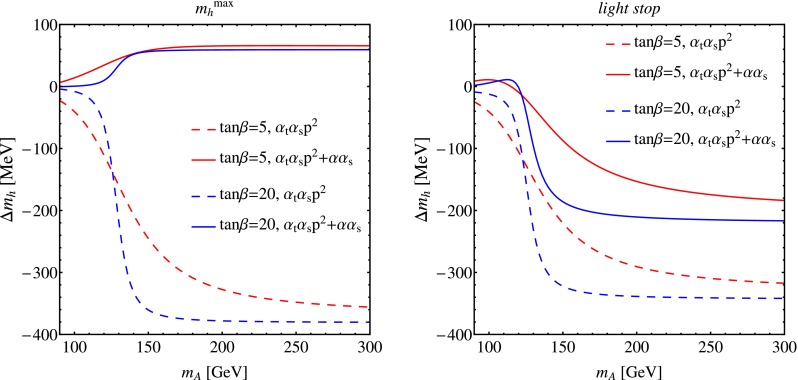
Corrections to the lightest-scalar mass as a function of ${{m}_{{\scriptscriptstyle A}}}$, for $\tan \beta =5$ (*red*) and for $\tan \beta =20$ (*blue*). The other MSSM parameters are chosen as in the $m_h^\mathrm{max}$ scenario (*left*) or as in the *light-stop* scenario (*right*). The *dashed lines* represent the contribution of the sole momentum-dependent part of the ${\mathcal {O}}(\alpha _{t}\alpha _{s})$ corrections, the *solid lines* include both the momentum-dependent ${\mathcal {O}}(\alpha _{t}\alpha _{s})$ corrections and the ${\mathcal {O}}(\alpha \alpha _{s})$ corrections

**Fig. 3:**
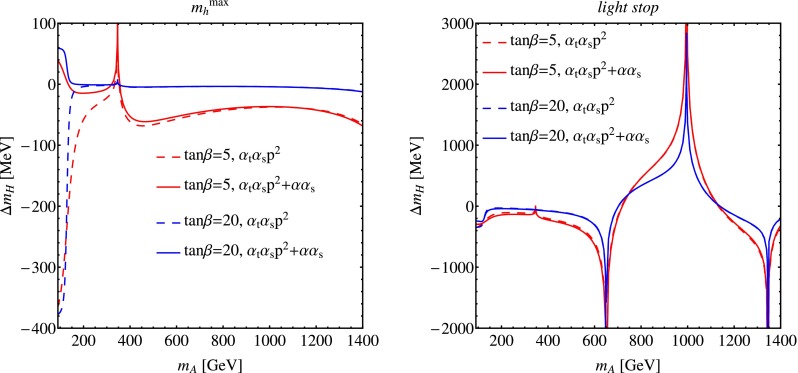
Same as Fig. 2 for the corrections to the heaviest-scalar mass

In Fig. 2 we illustrate the impact of the momentum-dependent ${\mathcal {O}}(\alpha _{t}\alpha _{s})$ corrections and of the ${\mathcal {O}}(\alpha \alpha _{s})$ corrections on the prediction for the lightest-scalar mass as a function of ${{m}_{{\scriptscriptstyle A}}}$, and in Fig. 3 we do the same for the heaviest-scalar mass. In each figure, the MSSM parameters for the left plot are chosen as in the $m_h^\mathrm{max}$ benchmark scenario of Ref. [77], while for the right plot they are chosen as in the modified *light-stop* scenario. In each plot, the dashed lines represent the contribution of the sole momentum-dependent part of the ${\mathcal {O}}(\alpha _{t}\alpha _{s})$ corrections, while the solid lines include both the momentum-dependent ${\mathcal {O}}(\alpha _{t}\alpha _{s})$ corrections and the ${\mathcal {O}}(\alpha \alpha _{s})$ corrections. The red lines were obtained with $\tan \beta =5$ while the blue lines were obtained with $\tan \beta =20$.

Figure 2 shows that the corrections to the lightest-scalar mass are negligible at low values of ${{m}_{{\scriptscriptstyle A}}}$, but they become larger and essentially independent of ${{m}_{{\scriptscriptstyle A}}}$ as the latter increases. The transition to this “decoupling” regime – where the lightest scalar has SM-like couplings and its mass is insensitive to the value of ${{m}_{{\scriptscriptstyle A}}}$ – is sharper for larger values of $\tan \beta$. In both the $m_h^\mathrm{max}$ and *light-stop* scenarios, the momentum-dependent ${\mathcal {O}}(\alpha _{t}\alpha _{s})$ effects decrease ${{m}_{h}}$ by 300–400 MeV at large ${{m}_{{\scriptscriptstyle A}}}$. However, as already seen in Fig. 1, the ${\mathcal {O}}(\alpha \alpha _{s})$ effects enter the prediction for ${{m}_{h}}$ with comparable magnitude but opposite sign, significantly reducing the total size of the correction.

Figure 3 shows that for low values of ${{m}_{{\scriptscriptstyle A}}}$, where the heaviest scalar is the one with SM-like couplings, the corrections to its mass are comparable to the ones that affect the lightest-scalar mass in the decoupling region. On the other hand, for larger values of the pseudoscalar mass – where ${{m}_{{\scriptscriptstyle H}}}\approx {{m}_{{\scriptscriptstyle A}}}$ – the corrections to the heaviest-scalar mass show a series of spikes, related to the opening of real-particle thresholds in diagrams that involve a virtual gluon. The first spike is visible in correspondence with ${{m}_{{\scriptscriptstyle A}}}= 2\,{{m}_{t}}$ in the plot on the left for the $m_h^\mathrm{max}$ scenario. More-pronounced spikes (note the different scale on the $y$ axis) are visible in correspondence with ${{m}_{{\scriptscriptstyle H}}}= 2\,m_{\tilde{t}_1}$, ${{m}_{{\scriptscriptstyle H}}}= m_{\tilde{t}_1}+m_{\tilde{t}_2}\,$, and ${{m}_{{\scriptscriptstyle H}}}= 2\,m_{\tilde{t}_2}\,$ in the plot on the right for the *light-stop* scenario. Analogous spikes would appear at larger values of ${{m}_{{\scriptscriptstyle A}}}$ in the $m_h^\mathrm{max}$ scenario, where the stops are heavier. We stress that our results are not reliable in the vicinity of these thresholds: the two-loop correction to the heaviest-scalar mass is actually divergent there, and the height of the spikes in the plots carries no physical meaning. A more sophisticated analysis, taking into account the widths of the virtual particles in the loops as well as non-perturbative QCD effects, would be necessary around the thresholds, but it is beyond the scope of our calculation.

Figure 3 also shows that, in the decoupling region and away from the thresholds, the corrections to the heaviest-scalar mass amount at most to a few hundred MeV, and they decrease in size with increasing $\tan \beta$. Moreover, the effect of the ${\mathcal {O}}(\alpha \alpha _{s})$ corrections is negligible (the dashed and solid lines are practically overlapping in the plots). Inspection of our analytic formulae shows that, in the decoupling limit, the ${\mathcal {O}}(\alpha \alpha _{s})$ corrections to ${{m}_{{\scriptscriptstyle H}}}$ are suppressed by one or two powers of $\tan \beta$, whereas the ${\mathcal {O}}(\alpha _{t}\alpha _{s})$ corrections contain unsuppressed contributions proportional to the square of the superpotential Higgs-mass parameter $\mu$. While corrections of this size might be considered negligible in comparison with the value of ${{m}_{{\scriptscriptstyle H}}}$ itself, they are not entirely irrelevant when compared to the difference ${{m}_{{\scriptscriptstyle H}}}-{{m}_{{\scriptscriptstyle A}}}$, which can be of the order of a few GeV and is the quantity of interest when a large physical mass for the pseudoscalar is taken as input in the calculation.

## Comparison with earlier calculations

The way we compute the ${\mathcal {O}}(\alpha _{t}\alpha _{s})$ and ${\mathcal {O}}(\alpha \alpha _{s})$ corrections to the entries of the inverse-propagator matrix for the neutral scalars allows for a relatively easy comparison with earlier calculations. We first renormalize all the relevant parameters in the $\overline{\mathrm{DR}}$ scheme, i.e. we introduce minimal counterterms that, by definition, subtract only powers of $1/\epsilon ,$ multiplied by coefficients that should be polynomial in the $\overline{\mathrm{DR}}$-renormalized masses and couplings. In a second step, we convert our results to the mixed OS–$\overline{\mathrm{DR}}$ scheme adopted in FeynHiggs, replacing the $\overline{\mathrm{DR}}$ top/stop parameters entering the one-loop part of the corrections with the corresponding OS parameters plus the finite one-loop shifts given in Ref. [25].

As a first obvious check, we took the limit of vanishing external momentum in the scalar self-energies entering the ${\mathcal {O}}(\alpha _{t}\alpha _{s})$ corrections and we compared our results with those in Ref. [25], finding full agreement. We also successfully compared the ${\mathcal {O}}(\alpha \alpha _{s})$ corrections at vanishing external momentum with the results of an independent calculation based on the effective-potential techniques of Ref. [25]. Note, however, that this comparison does not cover the ${\mathcal {O}}(\alpha \alpha _{s})$ contributions to the $Z$-boson self-energy. Concerning the latter, we checked that we can reproduce the result of Ref. [81] for the subset of two-loop diagrams that involve only quarks and a gluon, taking into account the fact that Ref. [81] employed dimensional regularization.

We then compared our results for the momentum-dependent corrections with those of Ref. [53], where the two-loop calculation of the Higgs masses was performed entirely in the $\overline{\mathrm{DR}}$ scheme. As mentioned in Sect. 1, the Higgs-mass corrections in Ref. [53] are organized in a different way with respect to our calculation, therefore we could compare only at the level of individual two-loop self-energies for scalars and pseudoscalars (the two-loop self-energy for the $Z$ boson was not computed in Ref. [53]). Rotating our scalar self-energies from the $(S_1,S_2)$ basis to the $(h,H)$ basis with the tree-level mixing angle defined in Ref. [53], we reproduce perfectly the results for the “top/gluon” and “top/stop/gluino” contributions to $\Delta \Pi _{hh}$ shown in figure 2 of that paper. This provides a full cross-check of the momentum-dependent ${\mathcal {O}}(\alpha _{t}\alpha _{s})$ contribution to the self-energy, as well as a partial check of the ${\mathcal {O}}(\alpha \alpha _{s})$ contribution, restricted to diagrams involving the stop squarks (the diagrams involving the other squarks are included in the “others” line in the above-mentioned figure). We also checked the analogous contributions to $\Delta \Pi _{h{\scriptscriptstyle H}}$, $\Delta \Pi _{{\scriptscriptstyle H}{\scriptscriptstyle H}}$, and $\Delta \Pi _{{\scriptscriptstyle A}{\scriptscriptstyle A}}$ against results provided by the author of Ref. [53], finding again perfect numerical agreement. Although our calculation and the one in Ref. [53] both use TSIL to compute the MIs, and thus cannot be considered entirely independent, the agreement in the results for the self-energies gives us confidence that the computation of two-loop Feynman diagrams in terms of MIs and the $\overline{\mathrm{DR}}$ subtraction of their divergences are correct in both papers.

Our results for the momentum-dependent ${\mathcal {O}}(\alpha _{t}\alpha _{s})$ corrections in the mixed OS–$\overline{\mathrm{DR}}$ scheme can in turn be compared with those of Ref. [56]. To start with, we compared our two-loop 1PI contributions to the scalar and pseudoscalar self-energies with analogous results provided by the authors of Ref. [56], and we found agreement within the accuracy of the sector-decomposition procedure used to compute the loop integrals in that paper. The successful comparison between two sets of self-energies in which the loop integrals were evaluated with TSIL and SecDec, respectively, validates the results for the two-loop MIs, thus reinforcing our cross-check of Ref. [53]. On the other hand, our results for the momentum-dependent ${\mathcal {O}}(\alpha _{t}\alpha _{s})$ corrections to the Higgs masses, obtained by combining the 1PI diagrams with all the necessary counterterm contributions, differ significantly from the ones in Ref. [56]. Considering for example the $m_h^{\mathrm{max}}$ scenario discussed in the previous section, we find that for large ${{m}_{{\scriptscriptstyle A}}}$ the lightest-scalar mass is subject to a negative correction of about 350–400 MeV (depending on $\tan \beta$; see the left plot in Fig. 2), whereas the corresponding correction in Ref. [56] is also negative but quite smaller, about 50–60 MeV (see the upper plot in figure 7 of that paper). We traced the reason for this discrepancy to an inconsistency in Ref. [56], related to the renormalization conditions for the Higgs fields and for $\tan \beta$.

In the $\overline{\mathrm{DR}}$ scheme, the WFR counterterm for each field $H^0_i$ can be related to the divergent part of the derivative of the scalar self-energy with respect to the external momentum:

18 $$\begin{aligned} \delta \mathcal{Z}^{(\ell )}_i = - \left[ \frac{d\, \mathrm{Re}\,\Pi ^{(\ell )}_{ii}(p^2)}{dp^2} \right] ^\mathrm{div}\quad (\ell =1,2). \end{aligned}$$

Indeed, when all parameters entering the one-loop part of the scalar self-energies are renormalized in the $\overline{\mathrm{DR}}$ scheme, Eq. (18) leads to the $\overline{\mathrm{DR}}$ WFR counterterms given in Eq. (9), in accordance with the anomalous dimensions of the Higgs fields given in Refs. [59, 60]. However, in the mixed OS–$\overline{\mathrm{DR}}$ scheme of Ref. [56] the top/stop parameters in the one-loop self-energies are renormalized OS. In that case, the use of Eq. (18) to determine the WFR counterterms leads to

19 $$\begin{aligned} \mathcal{Z}_2^{[56]}&= 1 - \frac{~\alpha _{t}^{{\mathrm{OS}}}}{4\pi }\,N_c\,\cdot \,\frac{1}{\epsilon }~+~ \frac{\alpha _{t}\alpha _{s}}{(4\pi )^2}\,2\,N_c\,C_F\nonumber \\&\cdot \left( \frac{1}{\epsilon ^2}-\frac{1}{\epsilon }\right) - \frac{\alpha _{t}}{2\pi }\,N_c\, \frac{\delta m_t}{m_t}\,\cdot \,\frac{1}{\epsilon }, \end{aligned}$$

where $\alpha _{t}^{{\mathrm{OS}}}$ is a scale-independent coupling extracted from the pole top mass, and $\delta m_t$ is the finite one-loop shift for the top mass given in Eq. (B2) of Ref. [25]. By converting the coupling $\alpha _{t}^{{\mathrm{OS}}}$ in the one-loop term of Eq. (19) into the corresponding $\overline{\mathrm{DR}}$ coupling, it is easy to see that $\mathcal{Z}_2^{[56]}$ differs from the $\overline{\mathrm{DR}}$ WFR constant in Eq. (9) by a finite two-loop term:

20 $$\begin{aligned} \mathcal{Z}_2^{[56]} = \mathcal{Z}_2^{\,{\overline{\mathrm{DR}}}} ~+~ \frac{\alpha _{t}}{2\pi }\,N_c\, \frac{\delta _\epsilon m_t}{m_t}, \end{aligned}$$

where $\delta _\epsilon m_t$ denotes the part proportional to $\epsilon$ in the top self-energy regularized with dimensional reduction:

21 $$\begin{aligned} \frac{\delta _\epsilon m_t}{m_t}&= \frac{\alpha _s}{4 \pi } \,C_F\, \left\{ -\frac{3}{2}\,\ln ^2 \frac{{{m}_{t}}^2}{\mu _{{\scriptscriptstyle R}}^2} +5\,\ln \frac{{{m}_{t}}^2}{\mu _{{\scriptscriptstyle R}}^2} - \frac{~\pi ^2}{4} - 9 - \frac{m_{\tilde{g}}^{2}}{{{m}_{t}}^2}\right. \nonumber \\&\times \left. \left( \frac{1}{2} \, \ln ^2 \frac{m_{\tilde{g}}^{2}}{\mu _{{\scriptscriptstyle R}}^2} - \ln \frac{m_{\tilde{g}}^{2}}{\mu _{{\scriptscriptstyle R}}^2} +\frac{~\pi ^2}{12} +1 \right) \right. \nonumber \\&+ \frac{1}{2}\left[ ~ \frac{m_{\tilde{g}}^{2}+{{m}_{t}}^2 - {m}_{\tilde{t}_1}^{2}- 2 \,{s}_{2\theta _{t}}\,m_{\tilde{g}}\, {{m}_{t}}}{{{m}_{t}}^2} \,B_\epsilon ({{m}_{t}}^2,m_{\tilde{g}}^{2},{m}_{\tilde{t}_1}^{2}) \right. \nonumber \\&+\left. \left. \,\frac{{m}_{\tilde{t}_1}^{2}}{{{m}_{t}}^2} \left( \frac{1}{2} \, \ln ^2 \frac{{m}_{\tilde{t}_1}^{2}}{\mu _{{\scriptscriptstyle R}}^2} - \ln \frac{{m}_{\tilde{t}_1}^{2}}{\mu _{{\scriptscriptstyle R}}^2} +\frac{~\pi ^2}{12} +1 \right) \right. \right. \nonumber \\&+\left. \left. (\tilde{t}_1 \rightarrow \tilde{t}_2, ~~ {s}_{2\theta _{t}}\rightarrow -{s}_{2\theta _{t}}) \right] \right\} . \end{aligned}$$

In the equation above $\mu _{{\scriptscriptstyle R}}$ is the renormalization scale associated to the Higgs WFR and to $\tan \beta$, while $B_\epsilon (s,x,y)$ denotes the coefficient of $\epsilon$ in the expansion of the Passarino–Veltman function $B_0$. An explicit expression for $B_\epsilon$ can be found, e.g., in Eq. (2.31) of the TSIL manual [52].

In the calculation of Ref. [56], where the top/stop parameters entering the one-loop part of the corrections are directly renormalized OS instead of being first renormalized in the $\overline{\mathrm{DR}}$ scheme and then converted to the OS scheme via a finite shift, the two-loop self-energies and tadpoles contain terms proportional to $\delta _\epsilon m_t$. Such terms would drop out of the final result for the renormalized inverse-propagator matrix if Eq. (20) was used to obtain the correct $\overline{\mathrm{DR}}$ definition for the WFR constant $\mathcal{Z}_2^{\,{\overline{\mathrm{DR}}}}$, and consequently for $\delta \tan \beta$, but they survive if the WFR constant is defined as in Eq. (19). To prove that these terms are indeed at the root of the observed discrepancies, we modified our own calculation, using $\mathcal{Z}_2^{[56]}$ – as obtained from Eq. (20) – instead of $\mathcal{Z}_2^{\,{\overline{\mathrm{DR}}}}$ and then computing a non-minimal counterterm for $\tan \beta$ via Eq. (10). We checked that, with this modification, we reproduce exactly the corrections to the renormalized inverse propagator shown in figures 5 and 10 of Ref. [56]. We also reproduce the corrections to the scalar masses shown in figures 7, 8, 12 and 13 of that paper, although small discrepancies persist in the case of the heaviest scalar when its mass is above the threshold ${{m}_{{\scriptscriptstyle H}}}=2\,m_t$. These residual discrepancies are formally of higher order in the perturbative expansion, and they result from different approximations in the two-step procedure for the determination of the poles of the propagator (namely, we drop the imaginary parts of the two-loop self-energies, while Ref. [56] keeps them).

In summary, we have found that in Ref. [56] the two-loop renormalization of the Higgs fields and of the parameter $\tan \beta$ is not performed in the $\overline{\mathrm{DR}}$ scheme as claimed in the paper, but rather in some non-minimal scheme where the WFR counterterms and $\delta \tan \beta$ differ from their $\overline{\mathrm{DR}}$ counterparts by finite, non-polynomial terms, and neither the Higgs fields nor $\tan \beta$ obey their usual renormalization-group equations (because of the explicit scale dependence of the additional terms). This inconsistency should be taken into account when comparing the results of Ref. [56] with those of calculations that actually employ $\overline{\mathrm{DR}}$ definitions for the WFR and for $\tan \beta$. First of all, to account for the difference in $\delta \tan \beta$, the input value for the $\overline{\mathrm{DR}}$-renormalized parameter $\tan \beta$ should be converted to the corresponding value in the non-minimal scheme of Ref. [56], according to

22 $$\begin{aligned} \tan \beta ^{[56]} = \tan \beta ^{\,{\overline{\mathrm{DR}}}} - \frac{\alpha _{t}}{4\pi }\,N_c\,\tan \beta ~ \frac{\delta _\epsilon m_t}{m_t}. \end{aligned}$$

However, Eqs. (5)–(7) show that the contributions of $\delta \tan \beta ^{(2)}$ to the entries of the Higgs mass matrix are suppressed by powers of $\cos \beta$. Consequently, the effect on the Higgs masses arising from a two-loop difference in $\delta \tan \beta$ is very small already for $\tan \beta =5$. In fact, the bulk of the numerical discrepancy between our results and those of Ref. [56] is due to higher-order effects that are directly related to the finite shift in the WFR. As discussed at the end of Sect. 2, such effects are included in the Higgs-mass corrections when the latter are computed in terms of loop-corrected Higgs masses and mixing, and can become numerically relevant when the loop-corrected masses differ significantly from their tree-level values.

## Conclusions

We computed the two-loop corrections to the neutral MSSM Higgs masses of ${\mathcal {O}}(\alpha _{t}\alpha _{s})$ and ${\mathcal {O}}(\alpha \alpha _{s})$ – i.e., all two-loop corrections that involve the strong gauge coupling when the only non-vanishing Yukawa coupling is $h_t$ – including the effect of non-vanishing external momenta in the self-energies. We relied on an IBPs technique to express the momentum-dependent loop integrals in terms of a minimal set of master integrals, and we used the public code TSIL to evaluate the latter. We obtained results for the Higgs-mass corrections valid when all parameters in the one-loop part of the corrections are renormalized in the $\overline{\mathrm{DR}}$ scheme, as well as results valid in a mixed OS–$\overline{\mathrm{DR}}$ scheme where the top/stop parameters are renormalized on-shell. Our results for the scalar and pseudoscalar self-energies in the $\overline{\mathrm{DR}}$ scheme confirm the results of an earlier calculation, Ref. [53], where they overlap. In addition, we obtained new results for the two-loop contributions to the $Z$-boson self-energy that involve the strong gauge coupling. The latter, which were not computed in Ref. [53], enter the ${\mathcal {O}}(\alpha \alpha _{s})$ corrections to the Higgs masses when the tree-level mass matrix of the scalars is expressed in terms of the physical $Z$-boson mass.

We also compared our results for the momentum-dependent ${\mathcal {O}}(\alpha _{t}\alpha _{s})$ corrections in the mixed OS–$\overline{\mathrm{DR}}$ scheme with those of a recent calculation of the same corrections, Ref. [56], and found disagreement. We traced the reason for the discrepancy to the fact that, contrary to what stated in Ref. [56], in that calculation the Higgs fields and the parameter $\tan \beta$ are renormalized in a non-minimal scheme instead of the usual $\overline{\mathrm{DR}}$ scheme. When this difference is taken into account, we reproduce the results of Ref. [56], providing in passing a cross-check of the codes used to evaluate the loop integrals in the two calculations (i.e., TSIL and SecDec, respectively). However, we noticed that TSIL, which implements dedicated algorithms for two-loop self-energy integrals, can be a thousand times faster than a multi-purpose code such as SecDec in the computation of the Higgs-mass corrections. This should be taken into consideration when including the momentum-dependent corrections in phenomenological analyses of the MSSM parameter space.

As to the numerical impact of the corrections computed in this paper, it could at best be described as moderate. We considered six benchmark scenarios compatible with the results of Higgs and SUSY searches at the LHC, and we found that both the momentum-dependent part of the ${\mathcal {O}}(\alpha _{t}\alpha _{s})$ corrections and the whole ${\mathcal {O}}(\alpha \alpha _{s})$ corrections can shift the prediction for the lightest-scalar mass ${{m}_{h}}$ by several hundred MeV. However, we noticed that – at least in the considered scenarios – the two classes of corrections enter the prediction for ${{m}_{h}}$ with opposite sign, and they compensate each other to a good extent. For what concerns the heaviest-scalar mass ${{m}_{{\scriptscriptstyle H}}}$, the impact of the new corrections is also modest, with the exception of regions around real-particle thresholds where a fixed-order calculation is not reliable anyway.

The predictions for the lightest-scalar mass, as obtained from popular codes for the determination of the MSSM mass spectrum, carry a theoretical uncertainty that has been estimated to be (at least) of the order of $\pm 3$ GeV – see, e.g., Refs. [78, 79] and the more recent discussion in Ref. [82]. Against this backdrop, the corrections presented in this paper can be considered sub-dominant, and their inclusion in public codes might seem less urgent than, e.g., the inclusion of the dominant three-loop effects [41, 42] or the proper resummation of large logarithms in scenarios with multi-TeV stop masses [39, 40, 82], both of which can shift the prediction for the lightest-scalar mass by several GeV. Nevertheless, one should not forget that the accuracy of the measurement of the Higgs mass at the LHC has already reached the level of a few hundred MeV – i.e., comparable to our sub-dominant corrections – and will improve further when more data become available. If SUSY shows up at last when the LHC operates at 13–14 TeV, the Higgs mass will serve as a precision observable to constrain MSSM parameters that might not be directly accessible by experiment. To this purpose, the accuracy of the theoretical prediction will have to match the experimental one, making a full inclusion of the two-loop corrections to the Higgs masses unavoidable. Our calculation should be regarded as a necessary step in that direction.

## Footnotes

1 We focus here on the MSSM with real parameters. Significant efforts have also been devoted to the Higgs-mass calculation in the presence of CP-violating phases, as well as in non-minimal supersymmetric extensions of the SM.

2 We define $\alpha _{t,b} = h_{t,b}^2/(4\pi )$, where $h_t$ and $h_b$ are the superpotential top and bottom couplings, respectively. We follow the standard convention of denoting by ${\mathcal {O}}(\alpha _{t})$ the one-loop corrections to the Higgs masses that are in fact proportional to $h_t^2 m_t^2$, i.e. to $h_t^4 v_2^2$. Similar abuses of notation affect the other one- and two-loop corrections.

3 The corrections to the Higgs masses involving the bottom Yukawa coupling could become relevant for large values of $\tan \beta$. When all parameters in the bottom/sbottom sector are renormalized in the $\overline{\mathrm{DR}}$ scheme, the ${\mathcal {O}}(\alpha _{b}\alpha _{s})$ corrections can be obtained from the corresponding results for the ${\mathcal {O}}(\alpha _{t}\alpha _{s})$ corrections via trivial replacements. On the other hand, an OS renormalization of the bottom/sbottom parameters would entail additional complications, as discussed in Refs. [27–29]. Anyway, the regions of the MSSM parameter space where the ${\mathcal {O}}(\alpha _{b}\alpha _{s})$ corrections to the Higgs masses are most relevant are being severely constrained by direct searches of Higgs bosons decaying to tau leptons at the LHC [61–64].

4 The renormalization of the Higgs fields and of $\tan \beta$ in the calculation of the Higgs-mass corrections was also recently discussed, in a somewhat different context, in Refs. [65, 66].

5 In the presence of infrared divergences associated to loops of massless quarks, the IBP reduction of the considered diagrams to MIs requires two additional functions. Their expression in terms of logarithms and polylogarithms can be obtained from the results of Ref. [76].

6 We omit a seventh scenario from Ref. [77], the so-called *low*-${{m}_{{\scriptscriptstyle H}}}$ scenario. Also, the parameters in the *light-stop* scenario are modified as in Ref. [80], to account for newer exclusion bounds from direct stop searches at the LHC.
